# Gastric cancer after laparoscopic adjustable gastric banding: A case report

**DOI:** 10.1016/j.ijscr.2024.109714

**Published:** 2024-04-27

**Authors:** Masaki Kanno, Akira Umemura, Shigeaki Baba, Haruka Nikai, Ryo Sugimoto, Akira Sasaki

**Affiliations:** a Department of Surgery, Iwate Medical University School of Medicine, 2-1-1 Idaidori, Yahaba-cho, Shiwa-gun, Iwate 028-3695, Japan; b Department of Molecular Diagnostic Pathology, Iwate Medical University School of Medicine, 2-1-1 Idaidori, Yahaba-cho, Shiwa-gun, Iwate 028-3695, Japan

**Keywords:** Bariatric surgery, Gastric cancer, Laparoscopic adjustable gastric banding, Metabolic surgery

## Abstract

**Introduction:**

Gastric cancer occurring after bariatric and metabolic surgeries is rare. We report a case of gastric cancer that developed at 14 years after laparoscopic adjustable gastric banding.

**Presentation of case:**

The patient was an obese 81-year-old woman who underwent LAGB at 14 years prior when her body mass index was 35.3 kg/m^2^. Anemia was noted during a visit to her family clinic. Subsequent esophagogastroduodenoscopy revealed a type 5 lesion (Macroscopic Classification of the Gastric Cancer in Japanese Classification of Gastric Carcinoma, The 15th Edition) near the greater curvature of the posterior wall of the gastric antrum. A biopsy indicated a poorly differentiated adenocarcinoma. Computed tomography showed no evidence of invasion of other organs, lymph node metastasis, or distant metastasis. The patient underwent laparoscopy-assisted distal gastrectomy, banding removal, Roux-en-Y reconstruction. The histopathological diagnosis was pT3N2M0 and pStage IIIA. The patient exhibited an uneventful postoperative course and was discharged on postoperative day 8. The patient has remained recurrence-free up to 12 months postoperatively.

**Discussion:**

While metabolic surgeries have been shown to reduce the risk of developing malignant diseases, including gastric cancer, the present patient developed gastric cancer at 14 years after laparoscopic adjustable gastric banding. The patient developed gastric cancer during a long-term course, indicating the importance of periodic examinations after metabolic surgery.

**Conclusions:**

Previous studies showed metabolic surgeries for obesity reduce the risk of developing malignancies, including gastric cancer; however, the present case suggests that gastric cancer may develop over a long-term course.

## Highlights

• Gastric cancer following laparoscopic adjustable gastric banding is rare.
• Gastric cancer following laparoscopic adjustable gastric banding can be safely treated laparoscopically.
• For patients at high risk of developing gastric cancer, consider periodic evaluation even after metabolic surgery.

## 1 Introduction

Metabolic surgeries for severe obesity have been shown to reduce the risk of malignant tumors, including gastric cancer. Roux-en-Y gastric bypass (RYGB) is commonly performed in Europe and the United States because of the low frequency of associated gastric cancer in these regions. In contrast, RYGB is not commonly performed in Japan because of the high incidence of gastric cancer in Eastern Asian people [1,2]. Additionally, gastric cancer occurring after metabolic surgeries, including laparoscopic adjustable gastric banding (LAGB), is rare [3]. Herein, we report a case of gastric cancer that developed 14 years after LAGB and was successfully resected using laparoscopy-assisted distal gastrectomy. We declare that the work has been reported in line with the updated SCARE guidelines [4].

## 2 Presentation of case

The patient was an obese 81-year-old woman who underwent LAGB (LAP-BAND®) at 14 years prior when her body mass index (BMI) was 35.3 kg/m^2^. The patient also had a medical history of esophagogastroduodenoscopy at 6 and 8 years prior, through which chronic gastritis and hyperplastic polyps were detected. At the time of her initial surgery, the patient tested positive for *Helicobacter pylori*, which was not eradicated. Other medical histories included cerebral infarction, abdominal aortic aneurysm, and appendicitis, for which underwent appendectomy. The patient smoked 15 cigarettes/day from 25 to 65 years of age, drank 100 mL/day of the Japanese liquor shochu (equivalent to approximately 3 units), and did not exhibit alcohol flush reaction.

**Fig. 1:**
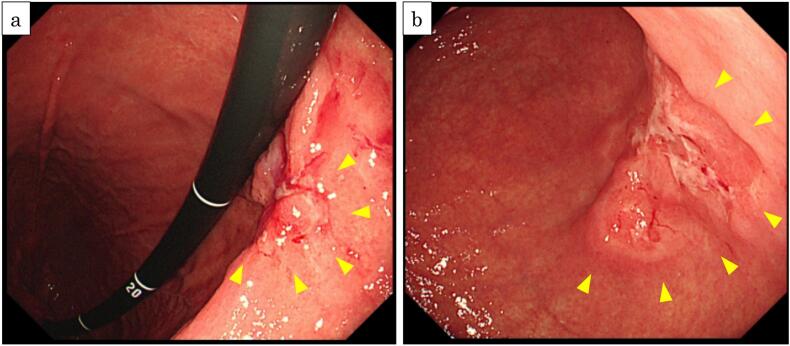
a: Preoperative esophagogastroduodenoscopy showed a type 5 lesion was found slightly posterior wall in the gastric angle and biopsy revealed a diagnosis of poorly differentiated adenocarcinoma., b: A type 2 lesion was found near oral side of the main lesion, and biopsy showed no evidence of malignancy.

Anemia was noted during a visit to her family clinic. Subsequently, esophagogastroduodenoscopy was performed, which revealed a type 5 lesion (Macroscopic Classification of the Gastric Cancer in Japanese Classification of Gastric Carcinoma, The 15th Edition) near the greater curvature of the posterior wall of the gastric antrum (Fig. 1a). A biopsy of the same area indicated a poorly differentiated adenocarcinoma. A type 2 tumor was further detected in the gastric angle (Fig. 1b); however, biopsy showed no malignant findings. Open-type atrophic gastritis was found, and no ulcerative changes or erosions during band formation were observed.

At the time of initial referral, blood test findings were as follows: red blood cell count, 3.87 10^6^/μL; hemoglobin, 10.4 g/dL; hematocrit, 33.7 %; and mild anemia. The levels of the tumor markers carcinoembryonic antigen (1.3 ng/mL) and carbohydrate antigen 19–9 (9.4 U/mL) were not elevated.

**Fig. 2:**
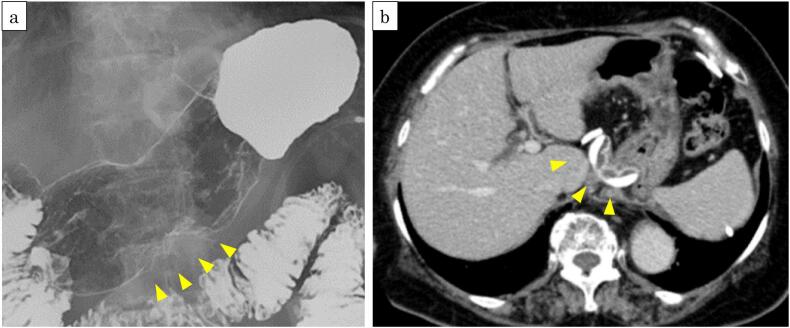
a: Upper gastrointestinal contrast imaging revealed a trapezoidal deformity mainly on the posterior wall., b: Computed tomography showed adjustable banding was observed in the upper gastric body. There was no obvious distant metastasis or lymph node metastasis.

Upper gastrointestinal contrast imaging revealed a trapezoidal deformity measuring 28.3 mm near the posterior wall of the lower gastric body. An adjustable banding was observed in the upper gastric body (Fig. 2a). Enhanced computed tomography showed no evidence of invasion of other organs, lymph node metastasis, or distant metastasis. An adjustable gastric banding was observed in the upper gastric body, with an implanted subcutaneous port in the left upper abdomen (Fig. 2b). Based on the forementioned findings, a diagnosis of gastric cancer (L, Less) with staging T2(MP) N0 M0, cStage I, and suspected gastric cancer (L, Less) with staging T2(MP) N0 M0, suspected cStage I, along with cholecystolithiasis was made. Laparoscopy-assisted distal gastrectomy, cholecystectomy, and gastric band removal were planned and performed after obtaining written informed consent. The patient had lost 18.2 kg from her initial metabolic surgery. Her BMI decreased from 35.5 kg/m^2^ to 28.6 kg/m^2^ at the timing of just before surgery.

**Fig. 3:**
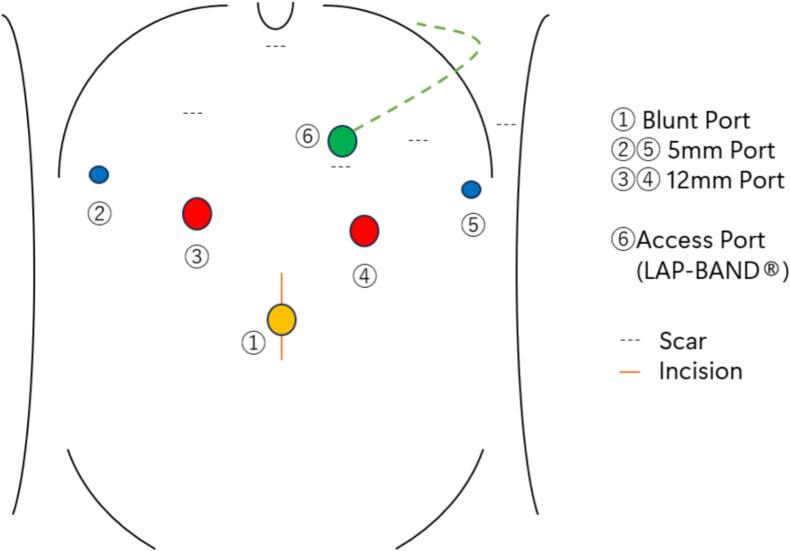
Port placement schema. The surgery was performed with 5 ports. The access port was located in the left upper abdomen.

**Fig. 4:**
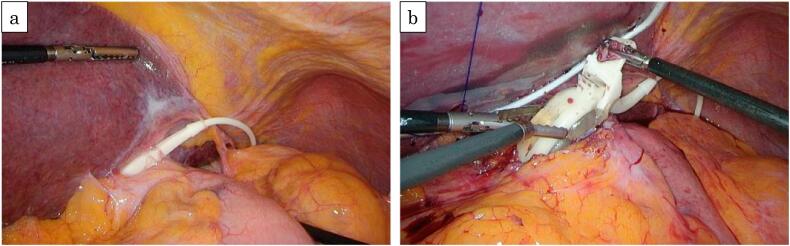
a: Operative findings: Adjustable banding showed adhesions to surrounding tissue., b: The tabs were grasped, and the banding was unlocked.

The patient was placed in lithotomy position under general anesthesia. Surgery was performed using the usual laparoscopy-assisted distal gastrectomy port placement (Fig. 3). For banding removal, the surrounding adhesions were dissected, and the tabs were grasped and unlocked with forceps. The catheter was detached after clipping, and the band and access ports were removed. The stomach at the banding site was left intact to prevent postoperative malnutrition. There was adequate proximal margin, and we considered removal of band improve intragastric pressure and mechanical stimulation. Distal gastrectomy, D2 lymphadenectomy, Roux-en-Y reconstruction and cholecystectomy were performed (Fig. 4a, b). The operative time was 5 h 59 min, and the amount of blood loss was 144 mL. The patient's postoperative course was uneventful. She started eating on postoperative day 3 and was discharged on postoperative day 8.

**Fig. 5:**
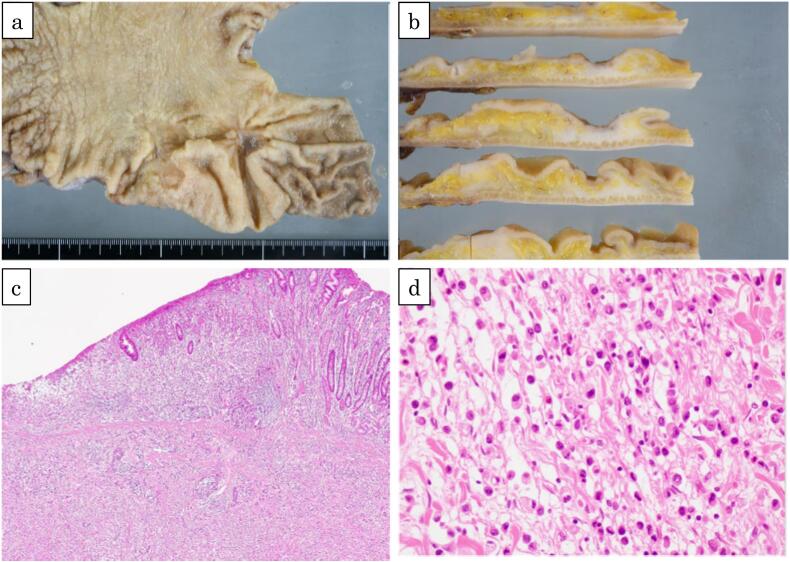
a: Macroscopic findings of the resected specimen. A 75 × 45 mm irregular raised depressed lesion was seen in the lower midline of the gastric body. The neighboring type 2 tumor was determined to be a series of lesions., b: Split face showed, a white lesion in the depressed area with a full wall., c: Histopathological findings. Microscopic examination revealed increased cell density in all layers of the ulcer (hematoxylin and eosin staining at ×40)., d: Higher magnification showed infiltrative proliferation of atypical cells with decreased connectivity and increased fibrous connective tissue with signet ring cell infiltration (hematoxylin and eosin staining at ×400).

Pathological examination revealed an irregular depressed lesion on the posterior wall of the lower part of the body (Fig. 5a, b). Histologically, the lesion was a poorly differentiated adenocarcinoma invading the subserosa with signet ring cell infiltration (Fig. 5c, d). The lesion was diagnosed as a series, with a neighboring lesion suspected to be gastric cancer. We did not employ immunohistochemistry stains because morphological features of poorly differenciated and signet ring cell adenocarcinoma were confirmed by specialized pathologists. The final histopathological diagnosis was pT3N2M0, corresponding to pStage IIIA (Union for International Cancer Control, 8th edition). The background gastric mucosa showed active gastritis and atrophy. However, *H. pylori* was not detected in the histopathological specimens.

Postoperative adjuvant chemotherapy with S-1 + docetaxel was administered, and the patient has remained recurrence-free up to 12 months postoperatively.

## 3 Discussion

LAGB is widely considered a relatively minimally invasive procedure, as it does not involve resection of the stomach or intestines. It has also become popular among laparoscopic metabolic surgeries at a relatively early stage. Nonetheless, the number of LAGB procedures performed has been decreasing over recent years. While metabolic surgeries have been shown to reduce the risk of developing malignant diseases, including gastric cancer, the present patient developed gastric cancer at 14 years after LAGB [5].

In addition to lowering the risk of developing obesity-related cancers, such as esophageal adenocarcinoma, renal cell carcinoma, gastroduodenal carcinoma, postmenopausal breast cancer, colon cancer, rectal cancer, liver cancer, pancreatic cancer, ovarian cancer and uterine cancer, metabolic surgeries have also been reported to reduce mortality [5]. Conversely, they have also been suggested to influence the development, diagnosis, and treatment of upper gastrointestinal malignancies, including esophageal and gastric cancers, as they cause anatomical changes in the stomach and contribute to the progression of esophageal reflux [3]. Performing endoscopic examination in the remnant stomach is difficult, particularly in RYGB, and some cases of advanced gastric cancer have been reported [6]. Banding of the stomach has been reported to cause reflux and stagnation of gastric contents and to be associated with Barrett's esophagus and a risk of esophageal cancer [7]. In some cases, edema, inflammation, and ulceration can be observed in the gastric mucosa below the band formation, accompanied by edema and dysplasia [8]. The presence of banding may cause increased intraluminal pressure, ischemia, and food friction [9]. Keeping the balloon at an appropriate size and location, performing frequent esophagogastroduodenoscopy follow-up, and administrating long-term proton pump inhibitors may contribute to the prevention of chronic gastritis in patients after LABG. The patient had a history of drinking and smoking, which are known to pose a significant risk of developing gastric cancer. Therefore, it is difficult to conclude that LAGB is a risk factor for the development of gastric cancer. However, as mentioned above, it is speculated that the presence of gastric banding may contribute to chronic gastritis.

In the present case, background pathological examination of the gastric mucosa revealed active gastritis and atrophy. *H. pylori* infection was noted before LAGB; however, eradication was not performed, and chronic active gastritis due to *H. pylori* infection was considered to have persisted thereafter. Gastric content stagnation and reflux due to the presence of banding were also considered to indicate chronic gastritis based on the pathological findings. It was difficult to differentiate between the former and latter based on pathological findings, and it was inferred that both were involved in the development of gastric cancer. There were many similar situations required eradication of *H. pylori* in patients with atrophic and/or chronic gastritis; therefore, prophylactic eradication of *H. pylori* had been covered by Japanese national health insurance system in 2013 [10]. Eradication of *H. pylori* is currently considered an important means of preventing the development of gastric cancer, so eradication should be offered to those infected with *H. pylori.*

Cases of gastric cancer in LAGB are rare, and a PubMed search for “adjustable gastric banding”, “gastric cancer” and “LAGB, gastric cancer” between 2008 and 2023 resulted in only 8 case reports, which we reviewed in detail (Table 1) [[11], [12], [13], [14], [15]]. The mean age of the patients in the report was 52.3 ± 14.9 years, with 3 males and 5 females. Symptoms included cardiac pain in 4 cases, vomiting in 2 cases, hematemesis in 2 cases, and anemia, abdominal pain, right-sided abdominal pain, hiccups, reflux symptoms, and abdominal distention in 1 case each. None of the patients exhibited specific symptoms requiring special mention. The mean duration from LAGB to the development of gastric cancer was 9.2 ± 6.3 years. This finding is consistent with another study that reported a mean duration of 8.6 years to the development of gastric cancer after other bariatric and metabolic surgical procedures were included [2]. The location of gastric cancer was noted to be variable. The occurrence of gastric cancer may be related to chronic mucosal irritation due to the stagnation of gastric acid or food in the stomach or to mechanical irritation of the stenotic area due to the presence of banding, as in some cases of gastric cancer occurring in the banding area [11]. However, in some reports, the localization of gastric cancer was found on the oral side of the banding, on the antral side, or even at some distance from the banding, as in the present case. In total, 5 of the 8 previously reported cases required resection, none of which were laparoscopically assisted.

**Table 1:** Summary of case report for gastric cancer following LAGB.

Reference	Age	Sex	BMI Before surgery	BMI at diagnosis of Tumor	Bariatric procedure	Reported symptoms	Tumor site	Time after bariatric surgery	Treatment
T. Hackert (2004) [11]	62	F	47.2	29.2	Open AGB	Epigastric pain, anemia	U, erosion with gastric cancer	10Y	None
C. Stroh (2008) [12]	63	F	46	36.7	LAGB	Hematemesis, urea	pouch above band	2Y	None
T. Szewczyk (2012) [13]	33	F	46.7	23.2	LAGB	Feeling of illness, mesogastrium pain, nausea	NR	5Y	OTG
G. Orlando (2014) [3]	37	F	NR	NR	AGB	Hematemesis	Less	6M	TG
A. Mangla (2018) [14]	50	M	NR	NR	LAGB	Acute pain in the right flank	M, Post	10Y	Chemotherapy
S. Eldar (2018) [9]	75	F	45.7	NR	LAGB	Chest pain, hiccups	Above band	16Y	OPG
S. Eldar (2018) [9]	39	M	40.7	NR	LAGB	Heart burn, regurgitation, epigastric pain, vomiting	NR	18Y	OTG
S. Eldar (2018) [9]	59	M	35	26.2	LAGB	Abdominal distension, epigastric pain, vomiting	NR	12Y	OSG
NS. Shetty (2020) [15]	67	M	NR	NR	AGB	NR	NR	NR	Chemotherapy
Our Case	81	F	35.3	28.1	LAGB	Anemia	M, Post	14Y	LADG

*AGB* Adjustable gastric banding, *LAGB* Laparoscopic adjustable gastric banding, *OTG* Open total gastrectomy, *TG* total gastrectomy, *OPG* Open proximal gastrectomy,

*OSG* Open subtotal gastrectomy, *LADG* Laparoscopy-assisted distal gastrectomy, *NR* No record.

Three cases of non-resection have been reported, all involving either metastasis or peritoneal dissemination. One case was treated with chemotherapy, and the other two with the Best Supportive Care. Among the initial symptoms, nausea and vomiting are common after metabolic surgeries, but it may be difficult for patients to recognize these as subjective symptoms. Weight loss becomes stationary after an average of one year post metabolic surgery, so decreasing weight in a long-term course after metabolic surgery can imply cachexia due to presence of malignant disease. As for the occurrence of gastric cancer after metabolic surgeries, it is thought that there are no specific subjective symptoms and that surveillance, including periodic upper gastrointestinal endoscopy, may contribute to detection of gastric cancer as time progresses postoperatively.

## 4 Conclusion

Prior studies have shown that metabolic surgeries for obesity reduce the risk of developing malignancies, including gastric cancer. However, the present case suggests that gastric cancer may develop over a long-term course. Unlike after other bariatric procedures such as RYGB, it is possible to perform a definitive gastrectomy with systematic lymph node dissection via minimally invasive approach after LAGB, furthermore, in regions with a high incidence of gastric cancer, LAGB and sleeve plus procedures may be preferable options.

## Abbreviations

RYGB: Roux-en-Y gastric bypass
LAGB: Laparoscopic adjustable gastric banding
CEA: Carcinoembryonic antigen
CA19–9: Carbohydrate antigen 19–9

## Ethical approval

The study protocol was approved by the institutional ethics committee of Iwate Medical University Hospital (approval number: MH2022-056) Yahaba-cho, Iwate Prefecture, Japan on 1 December 2022. Written informed consent was obtained from the patient analyzed in this study.

## Funding

The authors declare that they received no funding for this work.

## Guarantor

Masaki Kanno.

## Consent

Written informed consent was obtained from the patient for publication and any accompanying images. A copy of the written consent is available for review by the Editor-in-Chief of this journal on request.

## Research Registration Number

Not applicable.

## CRediT authorship contribution statement

MK, SB, and HN were responsible for the patient's clinical management and surgical procedure. RS made the pathological diagnosis and conducted the assessment. MK drafted the manuscript. AU and AS critically reviewed and discussed the content of the manuscript. All authors read and approved the final manuscript.

## Declaration of competing interest

The authors declare no competing interests.
